# Author response to comment on: Re-thinking benign inflammation of the lactating breast: Classification, prevention, and management

**DOI:** 10.1177/17455057231166452

**Published:** 2023-05-16

**Authors:** Pamela Douglas

**Affiliations:** School of Nursing and Midwifery, Faculty of Health, Griffith University, Brisbane, QLD, Australia

Dear Editor,

I am grateful to Dr Anne Witt and Dr Sheila Kredit for drawing attention to errors I have made in Appendix 1 of ‘Re-thinking Benign Inflammation of the Lactating Breast: Classification, Prevention, and Management’.^1^ I agree that accurate representation of research is of great importance, and I apologize for these mistakes.

Dr Witt and Dr Kredit are correct to state that I have falsely represented:

1. The rates of follow-up in the Witt et al.^2^ study and
2. Analysis of Witt et al. in Anderson et al.’s^3^ systematic review.

The Witt et al. study is not a randomized controlled study (RCT), but a nested case-control study. It is referred to as ‘quasi-experimental’ in the Anderson et al. systematic review, not as an RCT. In Appendix 1 of my article, I wrongly represent the numbers who responded to follow-up emails in the Witt et al. study, wrongly attributing these inaccurate numbers to Anderson et al. The Witt et al. study demonstrated excellent follow-up in the cohort who received Therapeutic Breast Massage in Lactation (TBML) and also in the control group.

Although debate is welcomed, and accurate representation of research essential, I nevertheless contend that TBML should not be recommended to breastfeeding women as evidence-based management of breast inflammation on the basis of Witt et al.’s study, for four reasons:

**1. TBML was delivered as one element in a complex breastfeeding intervention. Its efficacy was evaluated in small numbers for mastitis and plugged ducts, in the absence of a control group.**

TBML was delivered in the context of full breastfeeding support provided by an International Board Certified Lactation Consultant/registered nurse and/or breastfeeding medicine physician, which included latch correction, feeding patterns, antibiotic prescription, milk removal or analgesia as clinically indicated. The component of the study which investigates efficacy of TBML for mastitis and plugged ducts is a small, pre- and post-TBML assessment (mastitis n = 7, plugged ducts n = 17, see Supplement Appendix B), which lacks a comparison group. That is, pre- and post-intervention comparisons do not take into account the neurobiological effects of patient expectation (placebo effect), as Witt et al. acknowledge in their article.

**2. TBML did not show improvements in pain at 2-day and 12-week follow-up when the engorgement group was compared to the control group.**

Anderson et al. state in their analysis of Witt et al.,

Of the 15 participants with engorgement [in the TBML intervention group], measurements were taken from each breast, giving a total of 30 separate pain scores . . . These scores were treated independently (n = 30) in the pre-post analysis and combined (n = 15) for the comparison between the intervention and control groups, making interpretation quite difficult.

In the component of Witt et al. which investigates efficacy of TBML for engorgement, the intervention group (n = 15) was compared to a control group (n = 73); 47% of the intervention group had severe engorgement compared to 7% of the control group. Comparison of the engorgement intervention and control groups showed no meaningful difference in pain at day 2 nor in pain, exclusive breastfeeding or breastfeeding complications at week 12 in email follow-up.

**3. Pre- and post-TBML improvements can be explained by the ductal dilations (milk ejection) and milk removal components of TBML alone.**

TBML in the Witt et al. study achieves milk removal by alternating hand expression of milk with the massage technique, and by allowing direct breastfeeding of the infant during TBML (see Supplement Appendix A). The reduction in breast pain and also in size of plugged ducts observed immediately after TBML can be explained by the milk removal components of TBML alone, which are associated with milk ejections and ductal dilations.

**4. There is no pathophysiological model which explains the proposed efficacy of the gentle areola-to-axilla massage component of TBML.**

Is increased lymphatic drainage the proposed pathophysiological mechanism of light massage from the areola to the axillae? If so, this proposed mechanism isn’t supported by the latest research concerning the function of lymphatic vasculature. Interstitial fluid diffuses into the initial lymphatic capillaries in response to rising pressure gradients between breast stroma and lymphatic capillaries, which mechanically opens these capillaries. Lymphatic collection vessels contain valves, have smooth muscle in their walls, and are intrinsically contractile, actively pumping lymph towards the nodes. Although there is no convincing physiological rationale to support the belief that application of external pressure facilitates lymphatic removal of breast stroma interstitial fluid, there is reason to be concerned that an external pressure application which moves towards the axilla risks increased intra-alveolar milk pressures.

Various breast massage techniques are offered to breastfeeding women around the world, as Dr Witt and Dr Kredit note. Anderson et al. analyse the efficacy of a range of massage techniques in three RCTs and three quasi-experimental studies, including Witt et al.’s study of TBML. Although Anderson et al. conclude ‘Overall, different types of breast massage were reported as effective in reducing immediate pain for the participants’, I contend that neither Witt et al.’s data or Anderson et al.’s data support Therapeutic Breast Massage as an evidence-based intervention for presentations of lactation-related breast inflammation, despite its inclusion in Academy of Breastfeeding Medicine Clinical Protocol #36: The mastitis spectrum.^4^

Using the GRADE Working Group grades of evidence in their Summary of Findings, Anderson et al. report low certainty of outcomes for reduction in pain, increase in breast milk supply, and reduction or resolution of symptoms of breast inflammation, noting that ‘the true effect may be substantially different from the estimate of the effect’. Anderson et al. observe that the ability to replicate or generalize results of the six studies are limited by:

1. Significant heterogeneity of study methods, interventions and outcome measures
2. Lack of detailed explanation of breast massage techniques
3. Use of invalidated tools
4. Small sample sizes

Anderson et al. also note that requirement for extensive training for traditional Gua Sha^5^ and Oketani massage techniques,^6^ or requirement for seven consecutive days of massage combined with preparation of fresh topical cactus and aloe leaf lotion and pre- and post-massage application of aloe and cactus flesh, may not be practical in many settings.^7^

Because clinical breastfeeding support remains a research frontier,^8^ breastfeeding women are commonly referred to multiple providers for unproven interventions when problems emerge. Many popular treatments such as TBML lack both a convincing evidence-base and a robust underlying pathophysiological model. Such treatments may increase the financial burden for families and health systems, and raise the spectre of discriminatory breastfeeding support globally, with ease of access limited to affluent families in advanced economies.

Thank you for the opportunity to correct my mistaken representation of the Witt et al. and Anderson et al. studies in Appendix 1 of my article, for which I apologize. I welcome respectful discussion and debate concerning interpretation of existing studies and also the opportunity to amend errors, knowing that as clinicians and researchers we share the same commitment to improved outcomes for breastfeeding women and their babies.


Kind regards, Pamela Douglas


## Declarations

**Ethics approval and consent to participate:** Not applicable.

**Consent for publication:** Not applicable.

**Availability of data and materials:** Not applicable.
